# Global research trends on hypertension-induced myocardial fibrosis: A bibliometric analysis from 1976 to 2024

**DOI:** 10.1097/MD.0000000000045121

**Published:** 2025-10-24

**Authors:** Wei Zhao, Rongjie Tang, Yao Zhang, Yingxin Huo, Qiufang Lian

**Affiliations:** a Department of Cardiology, Xianyang Hospital of Yan’an University, Xianyang, Shaanxi Province, China.

**Keywords:** bibliometric analysis, hypertension, myocardial fibrosis, pressure, pulmonary hypertension

## Abstract

**Background:**

This study aimed to provide a thorough overview of research hotspots in hypertension-induced myocardial fibrosis (MF) through a bibliometric analysis approach.

**Methods:**

The Web of Science Core Collection database was searched for publications on hypertension-induced MF from 1976 to 2024. Bibliometric analysis and visualization were conducted by VOSviewer, CiteSpace, and Bibliometrix R package.

**Results:**

The study analyzed 1127 articles from 51 countries, with the USA and China leading in contributions. The volume of publications on hypertension-induced MF has grown annually. Key research institutions include Harvard University and Institut National De La Sante Et De La Recherche Medicale. The journal Hypertension is the most popular, while Circulation is the most cited. The research involves over 7249 authors, with Weber Kt having the highest publication count and international collaborations. The main research focuses are the pathogenesis and treatment strategies, with “pulmonary hypertension,” “pressure” and “ mechanisms” emerging as primary research hotspots.

**Conclusion:**

This bibliometric study provides an overview of research trends and advancements in hypertension-induced MF. Future research is expected to focus on noninvasive imaging methods, therapeutic targets and strategies, and left ventricular fibrosis associated with pulmonary arterial hypertension, aimed at improving diagnosis and treatment.

## 1. Introduction

Myocardial fibrosis (MF) is a fundamental pathophysiological component of various cardiovascular diseases.^[1]^ It is characterized by an excessive accumulation of extracellular matrix (ECM), resulting in increased ventricular wall stiffness and diminished systolic and diastolic function.^[2]^ The underlying mechanism involves the transformation of cardiac fibroblasts into myofibroblasts, which then proliferate and produce a substantial amount of ECM.^[3]^ MF can indeed be classified into 3 types based on histopathological changes: replacement fibrosis, interstitial fibrosis, and perivascular fibrosis, with the latter 2 often referred to collectively as reactive fibrosis.^[1,4]^ Fibrotic remodeling predominantly occurs within the myocardial interstitium, progressing insidiously through cumulative ECM deposition without substantial cardiomyocyte depletion. A paradigmatic example is systemic hypertension, wherein progressive interstitial and perivascular collagen accumulation elevates myocardial stiffness, ultimately manifesting as heart failure with preserved ejection fraction (HFpEF).^[1]^

Hypertension represents a significant global public health challenge and is a leading contributor to elevated morbidity and mortality from cardiovascular diseases.^[5]^ An estimated 8.5 million deaths were attributable to systolic blood pressure > 115 mm Hg, 88% of which were in low- income and middle-income countries.^[6]^ It disrupts myocardial compliance and induces hemodynamic pressure overload, which subsequently triggers fibrotic remodeling through transforming growth factor-β (TGF-β)/Smad3 activation.^[7,8]^ In hypertensive cardiomyopathy, reactive fibrosis characterized by type I/III collagen deposition is pathognomonically observed.^[9]^ This maladaptive remodeling progressively compromises both the architecture and contractile function of the left ventricle. MF associated with chronic hypertension is independently associated with substantially elevated risks of heart failure hospitalization, cerebrovascular accidents, and malignant arrhythmias.^[10–12]^ Recognizing MF as a preclinical pathophysiological hallmark of incipient heart failure is paramount for developing targeted prevention and precision treatment paradigms in cardiovascular medicine.^[13]^ Early detection enables timely initiation of guideline-directed therapies, potentially attenuating or even reversing fibrotic progression during the therapeutic window of opportunity.

Although there is growing interest in the classification of hypertension-induced MF,^[12,14]^ global research trend analysis on hypertension-induced MF remains scarce. Bibliometrics, formally introduced by Alan Pritchard in 1969,^[15]^ has gained prominence with advancements in computing technology and the internet. This quantitative field employs mathematical and statistical methods to analyze scientific publications, revealing patterns of contributions, research hotspots, and future trends within specific disciplines.^[16]^ Thus, this study aims to delineate global research trends, identify emerging hotspots, and explore cutting-edge directions in the field, thereby providing valuable insights to guide future research endeavors in hypertension-induced MF.

## 2. Methods

### 2.1. Search strategies and data collection

We conducted a literature search on hypertension-induced MF using the WoSCC from 1976 to 2024. The search formula used was: ((((((TS = (hypertension)) OR TS=(“high blood pressure”)) OR TS = (hypertony)) OR TS = (HPN)) OR TS = (HBP)) OR TS = (hyperpiesis)) OR TS = (hyperpiesia) AND (((((TS=(“cardiac muscle*”)) OR TS = (myocardia)) OR TS = (muscle*, cardiac)) OR TS = (muscle*, heart)) OR TS=(“heart muscle*”)) OR TS = (myocardium) AND (TS = (cirrhosis)) OR TS = (fibrosis). The publication language was restricted to English, and only articles were included. To prevent database update bias, the search was performed on November 5, 2024. All data were collected in text format, including publication and citation counts, titles, author information, institutions, countries, keywords, and journals for subsequent bibliometric analysis.

### 2.2. Bibliometric analysis and visualization

Several bibliometric tools were utilized for the statistical analysis, including Bibliometrix R package 4.3.3, VOSviewer 1.6.20, and CiteSpace 6.3.R1. Additionally, Microsoft Office Excel 2019 was also employed for the quantitative analysis of the data collected from these articles, allowing for a detailed examination of trends and patterns within the literature.

The Bibliometrix R package, created by Massimo Aria and Corrado Cuccurullo, is an open-source tool that generates comprehensive scientific maps of published literature, available on GitHub.^[17]^ In this study, Bibliometrix R package was utilized to quantitatively assess annual publication output, identify major journals, and predict future trends in cardiac fibrosis research. VOSviewer, a versatile software tool, was crucial for mapping collaborations, co-authorships, citations, and co-citations among institutions and authors.^[18]^ In our study, several analyses were performed utilizing the software, including country and institution analysis, journal and co-cited journal analysis, author and co-cited author analysis, and keyword co-occurrence analysis.

Meanwhile, CiteSpace was an information visualization software developed by Dr Chaomei Chen team.^[19]^ In this study, we utilized CiteSpace to conduct clustering, timeline, and burst analyses of co-cited references and co-occurring keywords, along with visualizing co-citation and co-authorship networks. Cluster labels were derived from keywords using the log-likelihood test (*P* < .001) and meticulously reviewed for accuracy. The timeline view effectively illustrates the evolution of various research areas, while several indicators analyze each cluster’s characteristics: temporal indicators like citation bursts highlight significant increases in citation frequency; structural indicators, including betweenness centrality, modularity (Q score), and silhouette score, provide insights into network organization. Betweenness centrality measures how often a node appears on the shortest path between others, indicating its influence, while the modularity score quantifies clustering strength, with values exceeding 0.3 indicating significant structures. Silhouette scores assess cluster quality, with scores above 0.3, 0.5, and 0.7 indicating increasing levels of homogeneity, plausibility, and credibility, respectively. Additionally, the Sigma indicator combines betweenness centrality and citation burst to assess a node’s overall impact, with higher Sigma values suggesting greater influence. In terms of network symbolism, the meaning of nodes and connecting lines aligns with that in VOSviewer, enabling a coherent interpretation of collaborative and co-citation relationships within the analyzed literature. In the visual analysis, the size of each node represents the number of associated publications, while the thickness of the lines connecting nodes reflects the strength of their relationships. Node colors may indicate different clusters or temporal aspects of the data.

To quantify the academic impact of individuals and journals, we utilized several metrics. The H-index, a key indicator for assessing a researcher’s academic contributions, offers insights into future performance. The G-index extends the H-index by evaluating citation distribution across a researcher’s publications, providing a more detailed view of citation impact.^[20]^ The G-index assesses citation distribution across a researcher’s publications, offering a detailed view of citation impact.^[21]^ The M-index, derived by dividing the H-index by the number of years since a researcher’s first publication, assesses research performance over time. An M-index below 1 suggests average performance, between 1 and 2 indicates above-average performance, and values above 2 signify exceptional impact.^[20]^

## 3. Results

**Figure 1.**
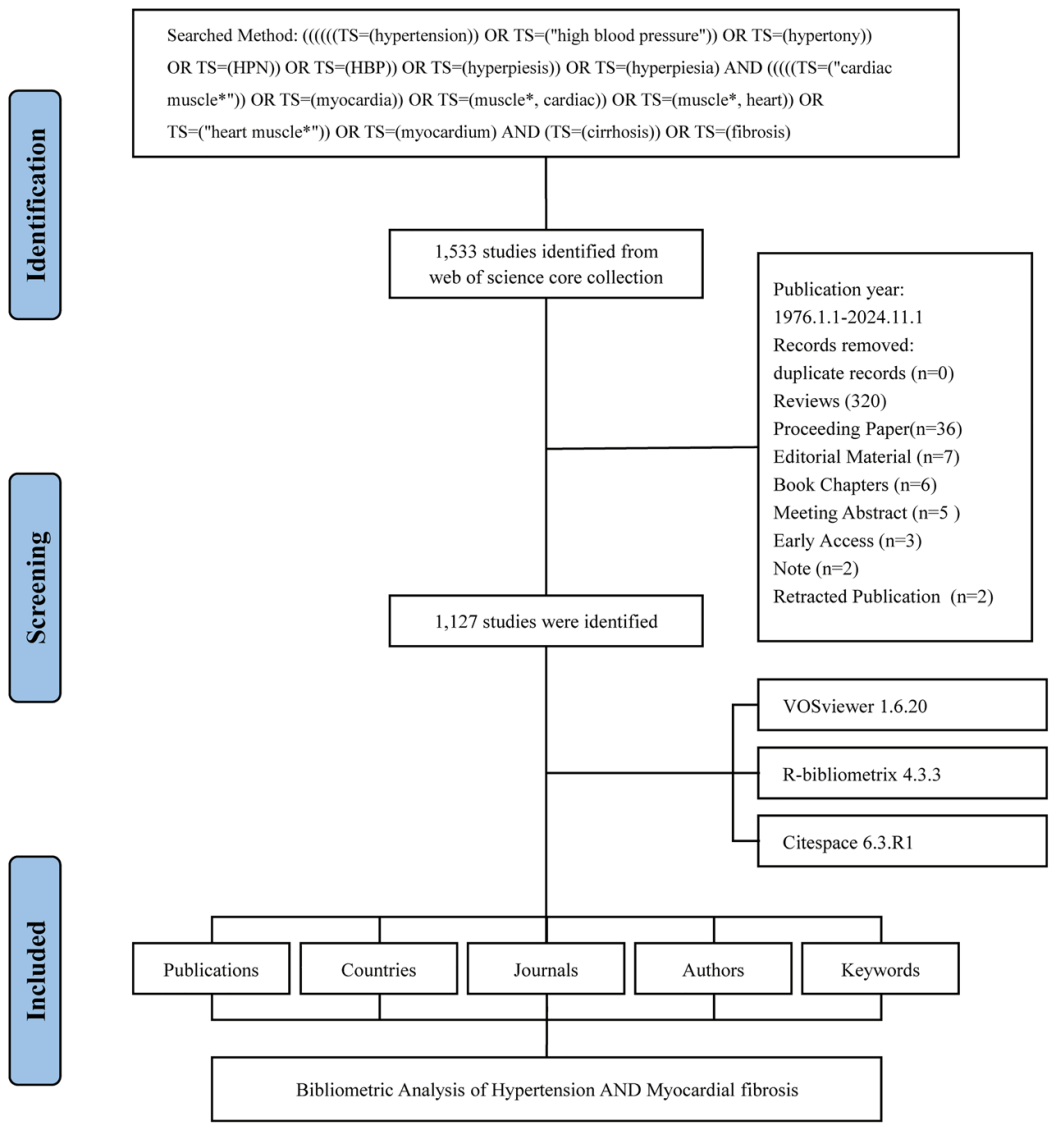
Flow chart of screening literatures.

**Figure 2.**
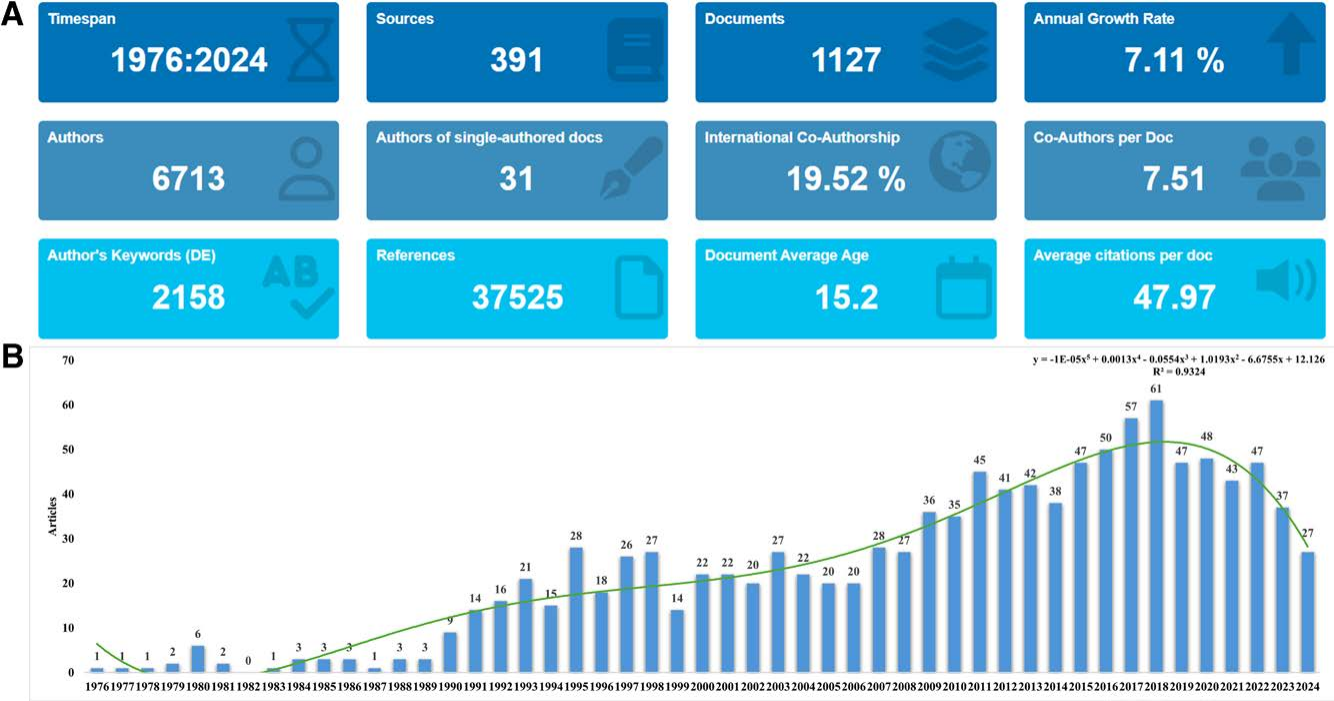
General information about the publications. (A) Overview of the publications; (B) annual publications from 1976 to 2024.

Based on the literature screening flow chart (Fig. 1), a total of 1127 articles were screened from 391 journals, showing an average annual growth rate of 7.11%. This body of research involved 7249 authors, with an average of 7.51 authors per article. The occurrence of single-author articles is relatively uncommon, comprising only 0.43% of the total. International collaboration was notably prevalent, with 19.52% of articles resulting from multinational partnerships. The average lifespan of these articles was 14.2 years, with an average citation count of 47.97 per article. The publication trend was delineated into 3 distinct phases (Fig. 2A). The first phase (1976–1989) was characterized by a lower volume of research, averaging 2.3 articles per year. The second phase (1990–2001) showed a marked increase, with an average of 21.09 articles per year. In the third phase (2002–2024), there was a first rising, then falling, with an average of 39.95 articles per year (Fig. 2B).

### 3.1. Contribution of countries/regions

**Figure 3.**
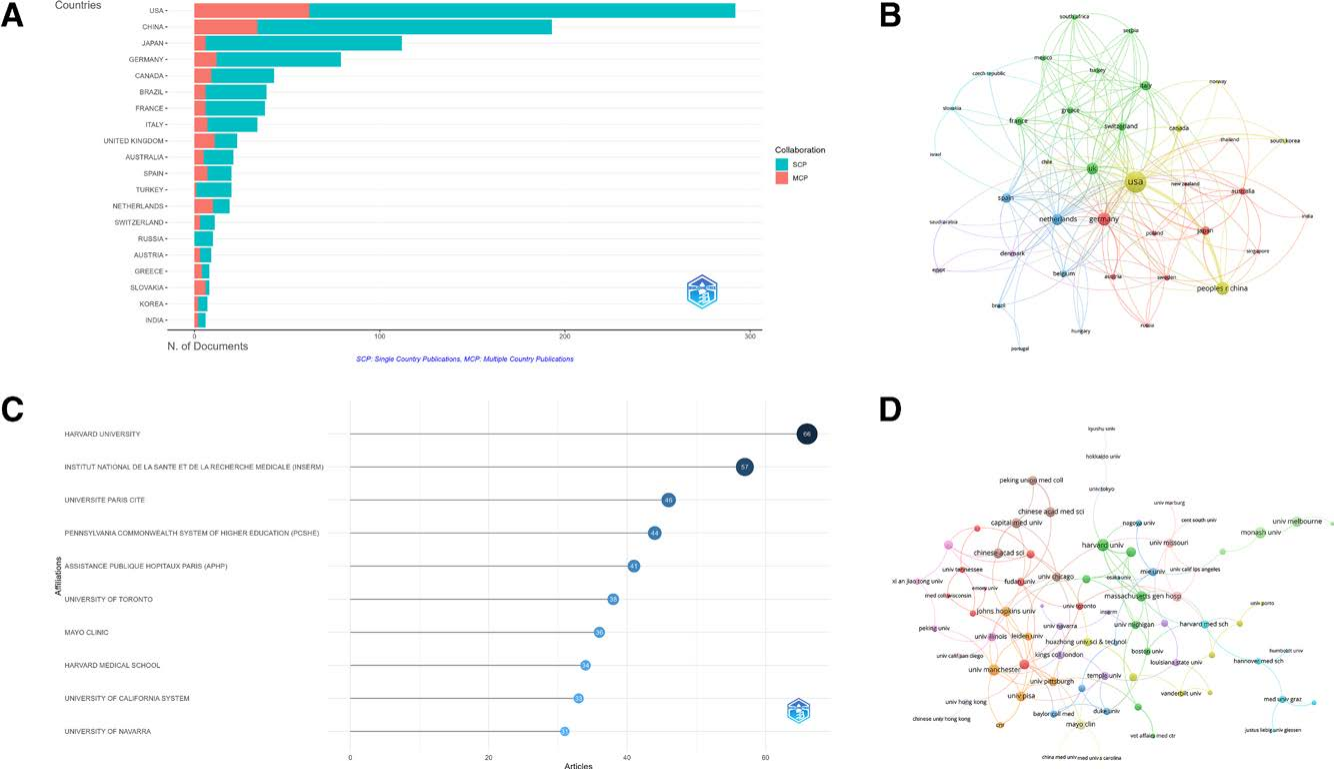
Analysis of countries and institutions. (A) Distribution of corresponding authors. The number of publications attributed to corresponding authors from different countries, distinguishing between SCP and MCP; (B) visualization map depicting the collaboration among different countries. The collaborative relationships between countries, with nodes representing countries, the size of nodes indicating publication count, and the thickness of links showing the strength of co-authorship collaborations; (C) top 10 institutions by article count and rank. The circle size shows the article count, with darker shades indicating higher ranks; (D) Visualization map depicting the collaboration among different institutions. Nodes represent institutions, with size indicating publication count. Links represent co-authorships, with thickness showing collaboration strength. Colors indicate different research clusters. MCP = multiple-Country publications, SCP = single Country publications.

The USA (514 articles), China (124 articles), Canada (79 articles), UK (73 articles) and Italy (73 articles) were the top contributors to hypertension-induced MF research (Fig. 3A). The USA not only led in publication volume but also in total citations (TC = 33,536), with an average of 65.2 citations per article. China, though second in publication output, had a lower citation rate (16 citations per paper), which was less frequently cited compared to the USA. European countries such as the UK (61.1 citations per paper) and American countries such as Canada (52.4 citations per paper) stood out for their high citation impact, despite producing fewer papers. In terms of global collaboration, the USA exhibited the highest total link strength (TLS) of 389, far surpassing the UK TLS of 191, which ranked second (Table S1, Supplemental Digital Content, https://links.lww.com/MD/Q349). Countries like Canada, South Africa, and Belgium also showed strong international collaborations, with China playing a central role in partnerships with the USA and other nations. The China (0.145) and the UK (0.613) had the highest proportions of multiple-country publications, reflecting strong international cooperation (Fig. 3B).

### 3.2. Institutions analysis

Among academic institutions, 6 of the top ten contributors were located in the USA, with Harvard University leading the ranking with 66 contributions, followed by the Institut National de la Santé et de la Recherche Médicale (INSERM) with 57 contributions (Fig. 3C). Other notable institutions included Université Paris Cité with 46 contributions, the Pennsylvania Commonwealth System of Higher Education with 44, and Assistance Publique - Hôpitaux de Paris (APHP) with 41 contributions. The institutional collaboration map (Fig. 3D) demonstrated that Harvard University, the University of Manchester, and Monash University function as central hubs for global research collaboration. These institutions not only generate a substantial volume of research but also actively participate in extensive international partnerships. Furthermore, INSERM, the University of Toronto, and Mayo Clinic are prominently involved in cross-border collaborations, further highlighting the global nature of research in this domain.

### 3.3. Journal analysis

**Figure 4.**
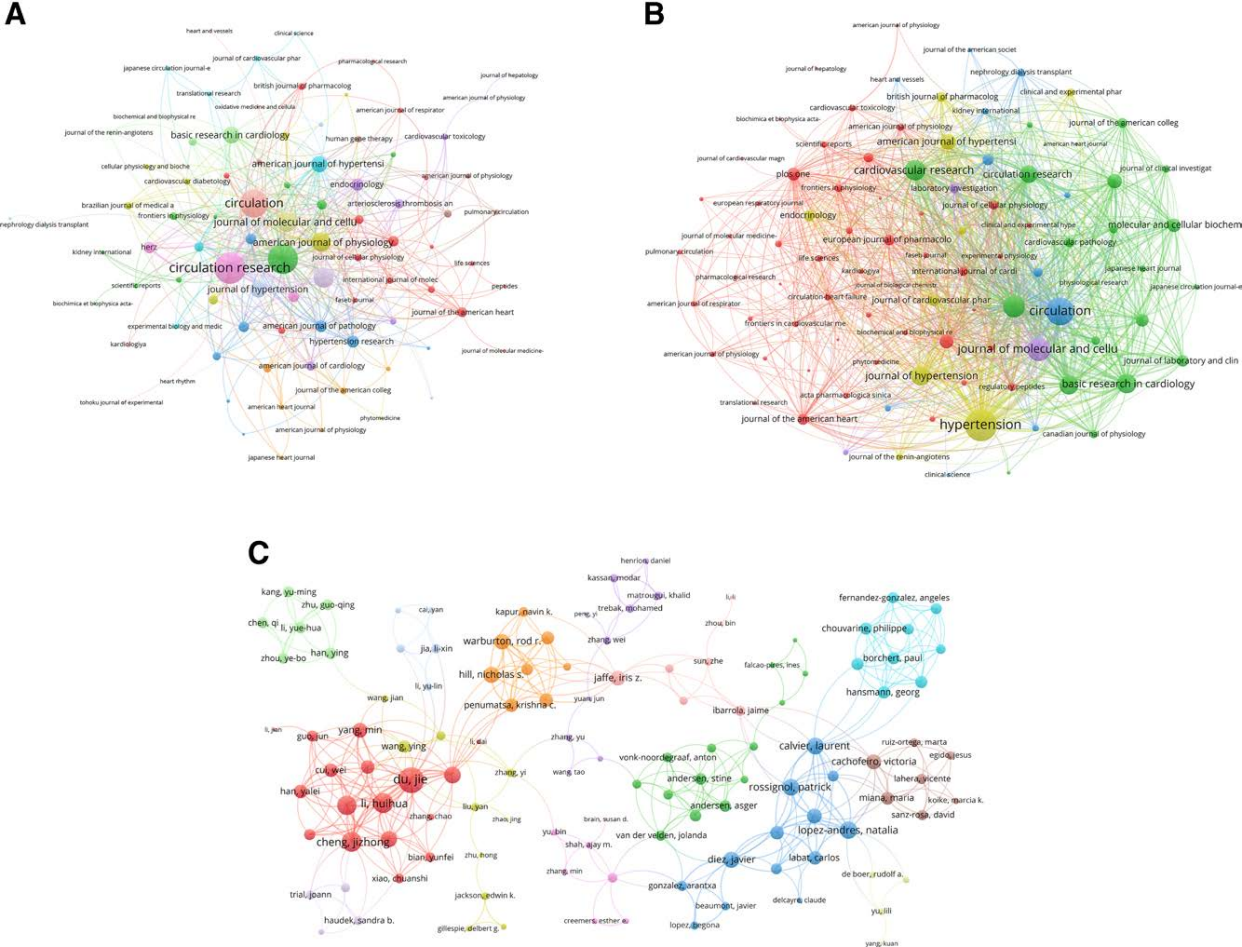
Analysis of journals and authors. (A) Distribution of publications in different journals. The frequency with which journals are cited together within the same articles reflects thematic or topical connections between the research they publish; (B) the network map of co-cited journals. The extent to which journals are linked is based on common references cited in their articles, indicating a shared intellectual foundation or research focus; (C) networks of authors. Nodes represent authors, with size indicating publication count. Links represent co-authorships, with thickness showing collaboration strength. Colors indicate different research clusters. Total link strength in collaboration networks measures the frequency of co-authorship between authors, indicating the level of collaborative research.

From 1976 to 2024, publications addressing hypertension-induced MF had appeared in 50 distinct journals. Analyzing these journals facilitated the identification of significant contributions within this field. Among the top 20 journals, 9 were based in the USA, exhibiting impact factors that range from 2.3 to 35.5, with an average impact factor of 7.645 (Table S2, Supplemental Digital Content, https://links.lww.com/MD/Q349). The journal Hypertension ranked first, with 74 papers (6.57% of the total), followed by Circulation with 40 papers and the American Journal of Physiology-Heart and Circulatory Physiology with 38 papers. The journal co-occurrence network encompassed 93 journals, each appearing at least 5 times (Fig. 4A). Co-citation analysis elucidates the relationships among various journals; the number of citations a journal receives serves as an indicator of its significance within a specific research domain, with a higher citation count signifying greater influence. The 3 leading journals, based on TLS, were Circulation Research (219), Hypertension (202), and Circulation (184). In the journal coupling network, which also included 93 journals with at least 5 connections (Fig. 4B), Hypertension led with a TLS of 6286, followed by Circulation with 4642 and the Journal of Molecular and Cellular Cardiology with 3174.

### 3.4. Author analysis

The top 20 most prolific authors and H-index were detailed in Table S3, Supplemental Digital Content, https://links.lww.com/MD/Q349. Over 7000 authors contributed to a total of 1127 publications. Weber KT emerged as the most prominent author, having published 31 articles and accumulating a TC of 5771. He was closely followed by Brilla CG, who had authored 21 publications and had a TC of 3480, making them the 2 most cited authors in this field. Among the 774 authors with more than 5 international collaborations, Weber KT leads with 78 collaborations, followed by Du Jie with 54 collaborations and Brilla CG with 49 collaborations (Fig. 4C).

### 3.5. Keyword co-occurrence and citation bursts analysis

**Figure 5.**
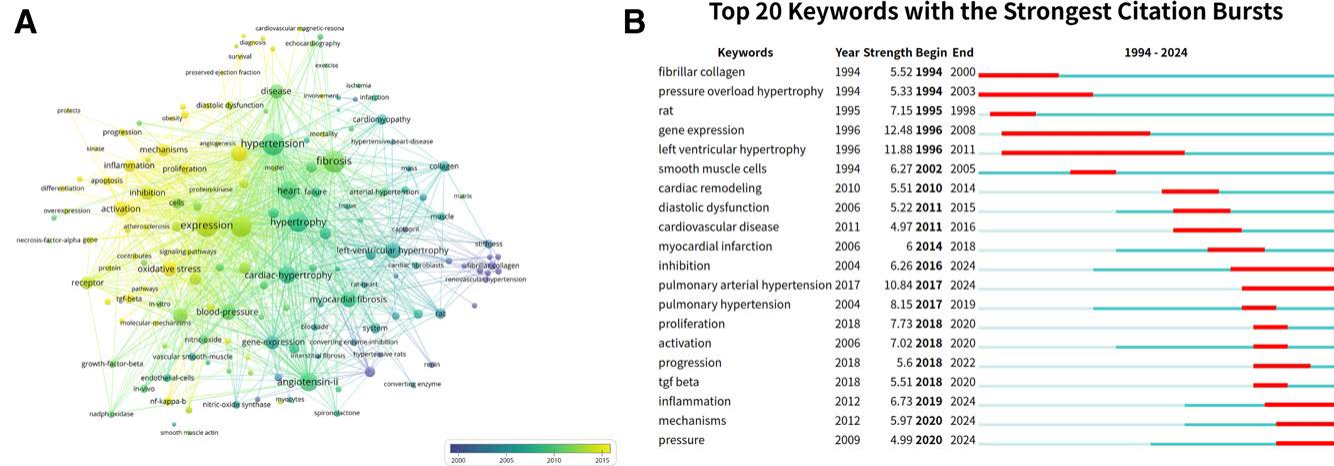
Analysis of keywords. (A) Visual analysis of keyword co-occurrence network analysis. This network visualization displays the co-occurrence of keywords in selected literature. Each node represents a keyword, with size indicating its frequency of occurrence. Links between nodes represent co-occurrence in the same documents, with thicker lines showing stronger associations. Colors reflect the average publication year of the articles, as indicated by the color gradient at the bottom right; (B) Top 20 keywords with the strongest citation bursts. The blue lines represent the period, and the red lines indicate the burst periods of the keywords.

Keywords provide a concise overview of a research paper’s topic, and analyzing their co-occurrence helps identify research hotspots and emerging frontiers related to hypertension-induced MF. We compiled a comprehensive list of the top 20 keywords ranked by frequency, which can be categorized into 3 primary research directions: clinical significance, risk factors, and pathophysiological mechanisms. Keywords associated with clinical significance include “heart failure” (183 occurrences), “dysfunction” (100), and “left-ventricular hypertrophy” (93). Those related to risk factors encompass “cardiac hypertrophy” (109). Mechanism-related keywords consist of “expression” (192), “angiotensin II” (122), “oxidative stress” (82), “activation” (82), “inhibition” (70), “receptor” (57), and “gene expression” (70) (Fig. 5A). Furthermore, the top 20 keywords exhibiting the most significant citation bursts as of 2024 provided insights into recent research trends and future potential (Fig. 5B). Notably, “gene expression” displayed the longest burst period from 1996 to 2008, whereas “pulmonary arterial hypertension (PAH)” has attracted considerable attention from 2017 to 2024. Keywords such as “inhibition,” “inflammation,” “mechanism,” and “pressure” were expected to remain prominent in scholarly discourse through 2024.

## 4. Discussion

This study conducted a bibliometric analysis of research articles pertaining to hypertension-induced MF. The findings reveal a consistent upward trend in annual publications on this topic. In addition to providing foundational insights, the analysis identified recent research hotspots and suggested potential emerging frontiers in the field. Notably, gene expression and left ventricular hypertrophy have been prominent research areas from the late 20th century into the early 2000s. Currently, research hotspots focus on diagnosis, correlations, apoptosis, and mechanistic investigations. Since 2002, the volume of research articles on hypertension-induced MF has increased significantly, with annual publications exceeding 20. The United States and China account for approximately 43% of these publications. Furthermore, 9 of the top twenty journals in this domain are based in the United States. The U.S. also leads in international collaboration and citation frequency, highlighting its substantial impact in this area of research. It is estimated that between 30% and 50% of American adults are affected by hypertension.^[22,23]^ Despite advancements in the diagnosis, treatment, and management of hypertension, only 54.4% of individuals affected achieve adequate blood pressure control.^[24]^ The established correlation between hypertension-induced fibrosis and the progression to heart failure likely accounts for the United States’ concentrated focus on research in hypertension-induced MF.^[12]^

The most cited article is titled “miR-133 and miR-30 regulate connective tissue growth factor: implications for a role of microRNAs in myocardial matrix remodeling,” published in the *Circulation Research* (IF_2023 = 16.4) in 2009, with a total of 719 citations.^[25]^ This study demonstrated connective tissue growth factor as attractive diagnostic marker and therapeutic target for abnormal myocardial matrix remodeling,^[26]^ which may be regulated by microRNA, such as miR-133 and miR-30. Additionally, miR-29a also was used as a circulating biomarker for hypertrophy and fibrosis.^[27]^ These results further show microRNAs have been increasingly suggested as biomarkers in cardiac remodeling. HFpEF is a common complication after fibrotic remodeling in myocardial infarction patients, showing dual roles of MF in this population.^[28]^ To date, most medication trials have shown neutral results for their primary endpoints, with only exercise training and weight loss demonstrating benefits for improving exercise intolerance and enhancing quality of life.^[29]^ The second most cited article, “Phenotype-Specific Treatment of Heart Failure with Preserved Ejection Fraction: A Multiorgan Roadmap,” was published in Circulation (IF_2023 = 35.5) in 2016, and has received 701 citations.^[30]^ This study recognizes HFpEF as a multisystem disorder involving the pulmonary circulation, right ventricular impairment, skeletal muscle dysfunction, and renal abnormalities, which opens a series of novel therapeutic targets requiring further validation.^[31]^ Notably, researchers have recognized an overlap between the metabolic conditions of skeletal muscle disorder and type 2 diabetes mellitus.^[32]^ Consistently, the third most cited article, “Evidence for cardiomyopathy in familial diabetes mellitus,” appeared in Journal of Clinical Investigation (IF_2023 = 13.3) in 1977, accumulating 673 citations,^[33]^ presenting collagen accumulation in perivascular loci and diffuse extravascular abnormality. Thus, widespread microvascular dysfunction may contribute to the development of diabetic cardiomyopathy.

### 4.1. Hotspots of hypertension-induced MF

Based on the findings from the keyword network and burst keyword visualization, the characteristics, focal points, and emerging trends in hypertension-induced MF research are summarized as follows:

Function-related keywords include heart failure, myocardial infarction, and dysfunction. Research indicates that extracellular remodeling and fibrosis resulting from hypertension impair the heart’s relaxation capabilities and elevate the risk of heart failure.^[34]^ Despite these findings, the clinical identification of fibrosis and subfibrosis remains challenging. Histological analysis is regarded as the gold standard for diagnosing MF; however, endomyocardial biopsy is an invasive procedure that is susceptible to sampling errors and is impractical for routine clinical use. As a result, there is a growing emphasis on noninvasive cardiac imaging techniques – such as echocardiography, single-photon emission computed tomography (CT), positron emission tomography, multi-slice CT, and cardiac magnetic resonance imaging^[35]^ – which are increasingly being developed and utilized. Importantly, considering the limitations of existing methods, artificial intelligence-guided electrocardiograms show promise for accessible and cost-effective detection of MF, suggesting that future research should prioritize prospective multicenter studies utilizing large, diverse clinical cohorts combined with cutting-edge machine learning architectures and robust external validation protocols.^[36]^

Mechanism-related keywords include angiotensin II (Ang II), oxidative stress, inflammation, and gene expression. The pathogenesis of MF remains incompletely understood. An expanding body of evidence underscores the role of various cellular effectors and signaling pathways in hypertension, as well as Ang II-induced cardiac fibrosis,^[37,38]^ oxidative stress,^[39,40]^ and inflammation.^[41]^ The complexity of signaling pathways, profibrotic mechanisms, and types of fibrosis indicates that a “one-size-fits-all” approach is unlikely to be effective for all heart diseases associated with fibrosis.^[42]^ Consequently, researchers are anticipated to continue exploring therapeutic targets and strategies for the treatment of MF.

Recent research has demonstrated a growing interest in left ventricular MF-related to PAH from 2017 to 2024. PAH is a serious condition associated with right heart failure and represents a significant global health challenge, adversely affecting overall health and quality of life.^[43]^ It is characterized by increased pulmonary vascular resistance and elevated pulmonary artery pressure, which can ultimately lead to right heart failure and mortality.^[44]^ The progression of pulmonary hypertension may result in right ventricular fibrosis, severely impairing or even obliterating right ventricular function.^[45]^ Pathological remodeling of the right ventricle is a critical factor in mortality among PAH patients, with myocardial cell apoptosis and metabolic disruptions playing significant roles in this process.^[46]^ Consequently, research has increasingly focused on strategies aimed at reducing cardiac afterload or directly protecting the structure and function of the right ventricle as potential treatments for PAH.

## 5. Future directions

Growing evidence indicates that early detection of hypertension-induced MF plays a pivotal role in cardiac functional recovery.^[47]^ Recently, cutting-edge multi-omics approaches and emerging biomarkers, including cell-free DNA and microRNAs, are revolutionizing MF-induced heart failure management strategies.^[48]^ Specially, recent breakthroughs in multi-omics research have significantly advanced our understanding of MF pathogenesis. Genomic investigations have uncovered disease-associated genetic variants and regulatory circuits governing fibrotic progression, while single-cell transcriptomic approaches have delineated heterogeneous cardiac fibroblast populations with specialized functional properties. Epigenetic analyses have demonstrated plasticity in chromatin states that regulate fibroblast phenotypic switching, complemented by proteomic discoveries of disease-relevant biomarkers and druggable targets. Metabolomic profiling has further elucidated profound disturbances in myocardial bioenergetics and metabolic flux during fibrotic remodeling, collectively providing a comprehensive framework for developing precision anti-fibrotic therapies, such as cell-type-specific interventions and metabolic modulators.^[49]^ These findings offer researchers novel insights to develop more personalized therapy for MF-related disorders.

## 6. Strengths and limitations

This study presents several notable advantages. First, it employs bibliometric methods for the first time to systematically analyze research on hypertension-induced MF, thereby offering valuable insights and comprehensive guidance for scholars in this field. Second, it utilizes 3 bibliometric tools, including VOSviewer and CiteSpace, both of which are well-established in bibliometric research, thereby enhancing the objectivity of the data analysis. Finally, bibliometric analysis provides a broader perspective on research trends and emerging topics compared to traditional review methods. However, the study also has certain limitations. First, the bibliometric analysis was conducted using the WoSCC database, which, while comprehensive, may have excluded relevant studies indexed in other databases such as PubMed or Scopus. This could introduce a selection bias and limit the generalizability of the findings. Second, the focus on English-language publications may result in an underrepresentation of research published in other languages, thereby influencing the results of geographic and thematic analysis. Future studies may benefit from a multi-database approach to enhance coverage and reduce selection bias.

## 7. Conclusion

This bibliometric analysis offers a thorough overview of the current research on hypertension-induced MF, highlighting key studies, emerging trends, and important collaborative networks. These insights are crucial for researchers and clinicians aiming to enhance their understanding and treatment of this significant aspect of cardiovascular health. Future research is expected to concentrate on the assessment of noninvasive imaging techniques, the exploration of therapeutic targets and strategies, and the investigation of left ventricular fibrosis related to PAH, with the goal of advancing diagnosis and treatment. Continuous bibliometric analysis will be essential for tracking research progress and guiding future inquiries.

## Author contributions

**Data curation:** Wei Zhao, Rongjie Tang, Yao Zhang, Yingxin Huo, Qiufang Lian.

**Formal analysis:** Wei Zhao, Rongjie Tang, Yao Zhang, Yingxin Huo, Qiufang Lian.

## Supplementary Material


