# Supplementary material for: Global research trends on hypertension-induced myocardial fibrosis: A bibliometric analysis from 1976 to 2024
Source: Medicine (Baltimore). 2025 Oct 24;104(43):e45121. doi: 10.1097/MD.0000000000045121 (PMC12558333; doi:10.1097/MD.0000000000045121)
Supplement: Supplementary file 1 [file medi-104-e45121-s001.docx]

**Table S1. Publication and citation profiles of leading countries.**

| **Country** | **Articles** | **Freq** | **MCP_Ratio** | **TP** | **TP_rank** | **TC** | **TC_rank** | **Average Citations** |
| --- | --- | --- | --- | --- | --- | --- | --- | --- |
| USA | 292 | 0.259 | 0.212 | 899 | 1 | 37110 | 1 | 59.4 |
| China | 193 | 0.171 | 0.176 | 636 | 2 | 9732 | 2 | 18.5 |
| Japan | 112 | 0.099 | 0.054 | 302 | 3 | 9702 | 3 | 36.8 |
| Germany | 79 | 0.070 | 0.152 | 286 | 4 | 7295 | 4 | 47.4 |
| Canada | 43 | 0.038 | 0.209 | 130 | 5 | 4001 | 8 | 33.1 |
| Brazil | 39 | 0.035 | 0.154 | 102 | 11 | 2164 | 12 | 25.5 |
| France | 38 | 0.034 | 0.158 | 95 | 9 | 4047 | 7 | 48.8 |
| Italy | 34 | 0.030 | 0.206 | 88 | 7 | 5442 | 5 | 77.7 |
| UK | 23 | 0.020 | 0.478 | 81 | 6 | 3495 | 10 | 52.2 |
| Australia | 21 | 0.019 | 0.238 | 59 | 10 | 3791 | 9 | 57.4 |
| Spain | 20 | 0.018 | 13 | 7 | 0.350 | 106 | 8 | 1364 |
| Turkey | 20 | 0.018 | 19 | 1 | 0.050 | 59 | 13 | 172 |
| Netherlands | 19 | 0.017 | 9 | 10 | 0.526 | 81 | 12 | 2603 |
| Switzerland | 11 | 0.010 | 8 | 3 | 0.273 | 47 | 14 | 392 |
| Russia | 10 | 0.009 | 10 | 0 | 0.000 | 31 | 18 | 15 |
| Austria | 9 | 0.008 | 6 | 3 | 0.333 | 38 | 15 | 454 |
| Greece | 8 | 0.007 | 4 | 4 | 0.500 | 26 | 19 | 99 |
| Slovakia | 8 | 0.007 | 2 | 6 | 0.750 | 21 | 22 | 236 |
| Korea | 7 | 0.006 | 5 | 2 | 0.286 | 20 | 24 | 241 |
| India | 6 | 0.005 | 4 | 2 | 0.333 | 12 | 33 | 112 |

Note(s): Articles: Publications of Corresponding Authors only. Freq: Frequence of Total Publications. MCP_Ratio: Proportion of Multiple Country Publications. TP: Total Publications. TP_rank: Rank of Total Publications. TC: Total Citations. TC_rank: Rank of Total Citations. Average Citations: The average number of citations per publication.

**Table S2. Bibliometric indicators of high-impact journals.**

| **Journal** | **H_index** | **IF_2023** | **JCR_Quartile** | **PY_start** | **TP** | **TP_rank** | **TC** | **TC_rank** |
| --- | --- | --- | --- | --- | --- | --- | --- | --- |
| Hypertension | 64 | 6.9 | Q1 | 1991 | 132 | 1 | 5468 | 2 |
| Circulation | 62 | 35.5 | Q1 | 1980 | 78 | 4 | 9295 | 1 |
| American journal of physiology-heart and circulatory physiology | 41 | 4.1 | Q1 | 1994 | 81 | 3 | 2297 | 6 |
| Cardiovascular research | 37 | 10.2 | Q1 | 1989 | 59 | 6 | 2740 | 5 |
| Journal of hypertension | 32 | 3.3 | Q1 | 1990 | 82 | 2 | 1809 | 11 |
| Journal of the american college of cardiology | 32 | 21.7 | Q1 | 1991 | 33 | 12 | 4282 | 4 |
| PloS ONE | 29 | 2.9 | Q1 | 2010 | 75 | 5 | 998 | 16 |
| Circulation research | 28 | 16.5 | Q1 | 1988 | 34 | 11 | 4439 | 3 |
| American journal of hypertension | 27 | 3.2 | Q2 | 1991 | 55 | 7 | 1128 | 14 |
| Journal of molecular and cellular cardiology | 27 | 4.9 | Q2 | 1979 | 39 | 10 | 2020 | 8 |
| Hypertension research | 23 | 4.3 | Q1 | 2001 | 52 | 8 | 565 | 34 |
| Journal of cardiovascular pharmacology | 21 | 2.6 | Q2 | 1991 | 39 | 9 | 663 | 25 |
| American journal of cardiology | 19 | 2.3 | Q2 | 1983 | 28 | 14 | 1874 | 10 |
| European journal of pharmacology | 17 | 4.2 | Q1 | 1997 | 24 | 17 | 384 | 47 |
| Journal of the american heart association | 16 | 5 | Q1 | 2012 | 32 | 13 | 368 | 48 |
| Circulation-heart failure | 15 | 7.8 | Q1 | 2009 | 19 | 22 | 593 | 33 |
| International journal of cardiology | 15 | 3.2 | Q2 | 1987 | 28 | 15 | 795 | 21 |
| Basic research in cardiology | 14 | 7.5 | Q1 | 1983 | 19 | 21 | 537 | 35 |
| Heart failure reviews | 14 | 4.5 | Q1 | 2005 | 16 | 28 | 336 | 58 |
| Cardiovascular pathology | 12 | 2.3 | Q2 | 1993 | 17 | 25 | 114 | 152 |

Note(s): H_index: The h-index of the journal, which measures both the productivity and citation impact of the publications. IF: Impact Factor, indicating the average number of citations to recent articles published in the journal. JCR_Quartile: The quartile ranking of the journal in the Journal Citation Reports, indicating the journal's ranking relative to others in the same field (Q1: top 25%, Q2: 25%-50%, Q3: 50%-75%, Q4: bottom 25%). TP: Total Publications. TP_rank: Rank of Total Publications. TC: Total Citations. TC_rank: Rank of Total Citations. Average Citations: The average number of citations per publication. PY_start: Publication Year Start, indicating the year the journal started publication.

**Table S3. Publication and citation profiles of high-impact authors.**

| **Authors** | **H_index** | **g-index** | **m-index** | **PY_start** | **TP** | **TP_Frac** | **TP_rank** | **TC** | **TC_rank** |
| --- | --- | --- | --- | --- | --- | --- | --- | --- | --- |
| Weber Kt | 35 | 43 | 0.95 | 1988 | 43 | 13.22 | 1 | 6657 | 1 |
| Brilla Cg | 23 | 25 | 0.66 | 1990 | 25 | 9.82 | 2 | 4174 | 2 |
| Diez Javier | 20 | 25 | 1.11 | 2007 | 25 | 3.43 | 3 | 1439 | 7 |
| Gonzalez Arantxa | 18 | 21 | 1.00 | 2007 | 21 | 1.96 | 5 | 1081 | 11 |
| Janicki Js | 17 | 18 | 0.46 | 1988 | 18 | 4.32 | 9 | 3103 | 3 |
| Lopez Begona | 17 | 18 | 0.94 | 2007 | 18 | 1.99 | 10 | 1011 | 12 |
| Díez J | 15 | 15 | 0.56 | 1998 | 15 | 2.57 | 12 | 1994 | 5 |
| Sowers James R. | 14 | 14 | 0.78 | 2007 | 14 | 1.34 | 19 | 974 | 13 |
| Lerman Amir | 13 | 20 | 0.72 | 2007 | 20 | 2.43 | 6 | 426 | 33 |
| Lerman Lilach O. | 13 | 21 | 0.72 | 2007 | 22 | 2.69 | 4 | 447 | 30 |
| Lin Yen-Hung | 13 | 20 | 0.77 | 2008 | 20 | 1.69 | 7 | 493 | 26 |
| Matsuoka H | 13 | 14 | 0.46 | 1997 | 14 | 2.48 | 17 | 434 | 32 |
| Carretero Oscar A. | 12 | 12 | 0.67 | 2007 | 12 | 1.60 | 23 | 812 | 15 |
| Habibi Javad | 12 | 12 | 0.67 | 2007 | 12 | 1.17 | 26 | 929 | 14 |
| Murohara Toyoaki | 12 | 14 | 0.63 | 2006 | 14 | 1.36 | 18 | 485 | 27 |
| Nagata Kohzo | 12 | 13 | 0.63 | 2006 | 13 | 2.13 | 21 | 391 | 37 |
| Rossignol Patrick | 12 | 15 | 0.80 | 2010 | 15 | 1.51 | 13 | 552 | 25 |
| Wu Vin-Cent | 12 | 19 | 0.71 | 2008 | 19 | 1.66 | 8 | 463 | 29 |
| Zannad Faiez | 12 | 15 | 0.80 | 2010 | 15 | 1.52 | 14 | 668 | 21 |
| Campbell Se | 11 | 13 | 0.32 | 1991 | 13 | 3.52 | 20 | 588 | 24 |

Note(s): H_index: The h-index of the journal, which measures both the productivity and citation impact of the publications. g_index: The g-index of the journal, which gives more weight to highly-cited articles. m_index: The m-index of the journal, which is the h-index divided by the number of years since the first published paper. TP: Total Publications. TP_rank: Rank of Total Publications. TC: Total Citations. TC_rank: Rank of Total Citations. Average Citations: The average number of citations per publication. PY_start: Publication Year Start, indicating the year the journal started publication.
